# Less is more: repellent-treated fabric strips as a substitute for full screening of open eave gaps for indoor and outdoor protection from malaria mosquito bites

**DOI:** 10.1186/s13071-022-05384-7

**Published:** 2022-07-20

**Authors:** Margaret Mendi Njoroge, Alexandra Hiscox, Adam Saddler, Willem Takken, Joop J. A. van Loon, Ulrike Fillinger

**Affiliations:** 1 International Centre of Insect Physiology and Ecology (icipe), Human Health Theme, P.O. Box 30772-00100, Nairobi, Kenya; 2 Wageningen University & Research, Laboratory of Entomology, P.O. Box 16, 6700 AA Wageningen, The Netherlands; 3 ARCTEC, London School of Hygiene & Tropical Medicine, Keppel Street, London, WC1E 7HT UK; 4 Department of Epidemiology and Public Health, Swiss Tropical and Public Health Institute, Socinstrasse 57, 4051 833 Basel, Switzerland; 5 Ifakara Health Institute, P.O. Box 74, Bagamoyo, Tanzania; 6 Malaria Atlas Project, Telethon Kids Institute, 15 Hospital Ave, Nedlands, Perth, WA 6009 Australia

**Keywords:** Transfluthrin, Eave screens, Eave strips, Malaria control, Spatial repellent, House screening, Human landing catches

## Abstract

**Background:**

Providing protection from malaria vector bites, both indoors and outdoors, is crucial to curbing malaria parasite transmission. Screening of house entry points, especially with incorporated insecticides, confers significant protection but remains a costly and labour-intensive application. Use of spatial repellents has shown promise in creating areas of protection in peri-domestic areas.

**Methods:**

This study aimed at comparing the protection provided by transfluthrin-treated and untreated complete screens over open eave gaps with incomplete transfluthrin-treated eave strips as a potential replacement for a full screen. Human landing catches were implemented independently inside and outside an experimental hut under controlled semi-field conditions, with insectary-reared *Anopheles arabiensis* mosquitoes.

**Results:**

The odds of a female mosquito finding a human volunteer indoors and attempting to bite were similar whether the eaves were completely open or there was an untreated fabric strip fixed around the eaves. However, when the eave gap was completely screened without insecticide, the odds of receiving a bite indoors were reduced by 70% (OR 0.30, 95% CI 0.20–0.47). Adding transfluthrin to the full screen, further increased the protection indoors, with the odds of receiving a bite reduced by 92% (0.08, 95% CI 0.04–0.16) compared to the untreated screen. Importantly, the same protection was conferred when only a narrow transfluthrin-treated fabric strip was loosely fixed around the eave gap (OR 0.07, 95% CI 0.04–0.13). The impact of the transfluthrin treatment on outdoor biting was correlated with evening temperatures during the experiments. At lower evening temperatures, a transfluthrin-treated, complete screen provided moderate and variable protection from bites (OR 0.62, 95% CI 0.37–1.03), whilst at higher evening temperatures the odds of receiving a bite outdoors was over four times lower in the presence of transfluthrin, on either a full screen (OR 0.22 95% 0.12–0.38) or a fabric strip (OR 0.25, 95% 0.15–0.42), than when no treatment was present.

**Conclusion:**

The findings suggest that transfluthrin-treated fabric strips can provide a substitute for complete eave screens. They are a simple, easy-to-handle tool for protecting people from malaria mosquito bites indoors and potentially around the house in climatic areas where evening and night-time temperatures are relatively high.

## Background

Most malaria-endemic areas in sub-Saharan Africa are rural, where housing structure pre-disposes people to mosquito bites, because of either open gaps along the roofs’ eaves or unscreened windows and doors. Rural housing also often necessitates evening activities like cooking to be conducted outdoors or in makeshift kitchen huts near the main house that do not offer any protection from mosquito bites [1–4].

Eave gaps provide ventilation indoors, and sealing them off can lead to reduced air flow and potentially increased indoor temperatures unless additional house adjustments are done to remedy this [5, 6]. Improved housing has been shown to significantly reduce house entry of adult mosquitoes and malaria infections [6–10]. Screening of open eave gaps in combination with screening of doors and windows is recommended for effective reduction of mosquito house entry [10]. Despite the advantages of eave screening, a major challenge in this approach is the availability of low-cost materials, as well as the difficulty in properly fixing barriers in existing structures which may have uneven surfaces [11, 12]. There is also a concern that screening the house may reduce adherence to the use of long-lasting insecticide-treated bed nets (LLINs) because of perceived reductions in biting pressure [12], which could lessen the protection received by occupants and needs to be addressed in additional education campaigns.

In situations where house improvement is not immediately achievable for varied reasons, and where LLINs provide incomplete protection against malaria infection, alternate means of vector control should be added to provide protection to persons when they are in the outdoor periphery of their homes or indoors but not yet under protective bed nets [13]. Evening and outdoor mosquito bitings have been cited as contributors to the rising importance of residual malaria transmission that remains unaddressed [14–17].

Spatial repellents have received increasing attention in recent years as complementary control tools against adult malaria mosquitoes [18–25]. Unlike toxicants that result in mortality of mosquitoes or irritants that result in agitation, spatial repellents drive mosquitoes away from a treated space, thereby being proposed as ideal candidates for outdoor protection [24, 26]. In addition, pyrethroid spatial repellents have been shown to induce deterrence, even in mosquitoes which are resistant to this group of insecticides [26, 27]. As a deterrent mode of action may not lead to complete toxic effects, the selection pressure exerted by spatial repellents might be lower than that exerted by toxicants which end up selecting resistant mosquitoes in the population by killing of the more susceptible ones [26, 28]. An added advantage of spatial repellents is the possibility to exert repellency on a wide range of mosquito species, therefore potentially disrupting not just the transmission of malaria, but other mosquito-borne diseases such as dengue, chikungunya and Zika virus among others [18, 29, 30]. Additionally, spatial repellents can be highly effective against biting by nuisance mosquitoes.

One such candidate spatial repellent is transfluthrin, which is a fast-acting, low persistence, 15-carbon pyrethroid insecticide. It has been included in mosquito repellent and killing products such as mosquito coils and vaporizers [22, 31–35]. Release of transfluthrin on commercial products relies on an external source of energy or combustion and has many limitations: coils produce smoke and need to be actively managed for optimal protection [36]; electrical emanators are not affordable for most rural low-income communities and require a power socket in the area to be protected [37]. Non-powered, low maintenance passive release options would make this intervention more accessible for large-scale use [20, 37, 38].

Inclusion of the repellent on material used for eave screening for passive release could increase the protection conferred indoors by existing tools and could potentially allow peri-domestic release of the repellent thereby also providing the much-needed bite protection outdoors, even against pyrethroid-resistant malaria vectors [22, 39–42]. Recent work done in Kenya and Tanzania has explored the use of insecticide-treated strips that are secured on traditional houses to achieve a protective effect against malaria mosquitoes instead of the use of full eave screening [20, 25, 39]. The idea of using a repellent-treated fabric strip that does not fully cover the eave gaps offers possible cost and logistical advantages as there would be less material used and less precise fitting necessary compared to the treated or untreated complete screens aiming to cover the entire gap [8, 12].

The present study aimed at comparing the protection provided by both treated and untreated complete screening and incomplete screening with treated and untreated eave strips against bites from insectary-reared *Anopheles arabiensis* (Mbita strain, Kenya) under controlled semi-field conditions. Human landing rates, both indoors and outdoors, were measured as a basis for determining if a transfluthrin-treated eave strip would be a suitable replacement for a full eave screen (treated or untreated).

## Methods

All experiments were conducted at the International Centre of Insect Physiology and Ecology at the Thomas Odhiambo Campus (icipe-TOC) in Mbita, on the shores of Lake Victoria in Homabay County, western Kenya (0°26′06.19"S, 34°12′53.13"E; altitude 1137 m).

### Semi-field systems

**Fig. 1:**
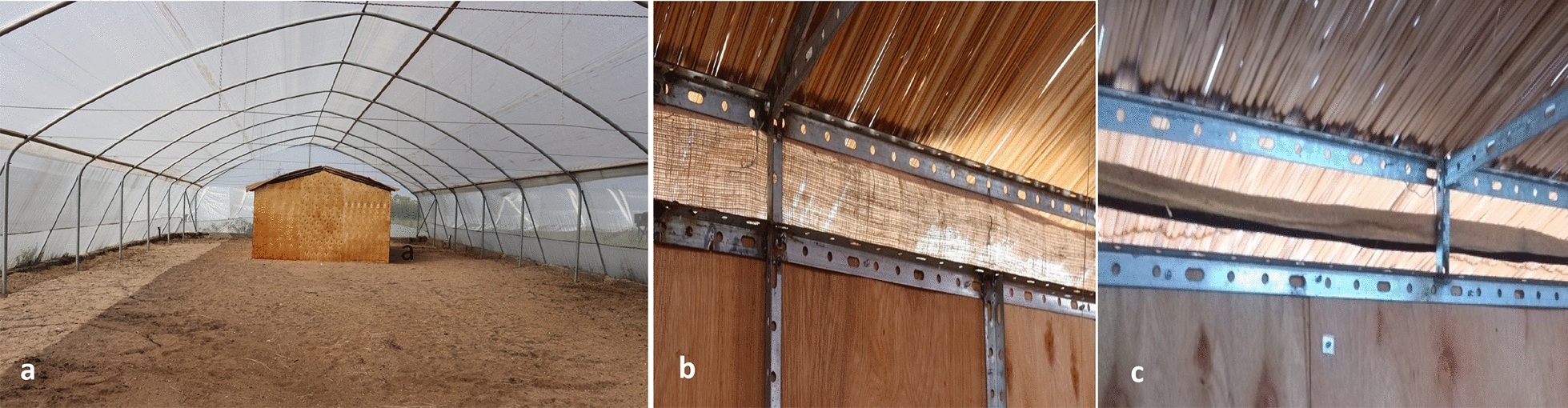
**a** Pictorial presentation of a semi-field system (27 m × 11 m), including an experimental hut made of plywood with a grass thatched roof (6.5 m × 3.5 m). **b** Application of an eave screen secured on the eave gap of the experimental hut using aluminium wires to ensure complete coverage of the eave gap (**c**) and eave strip secured on the eave gaps of the experimental hut using aluminium wires to ensure equal distance (2.5 cm) above and below the eave fabric

Two mesh-screened greenhouses (Amiran Kenya Ltd, Nairobi, Kenya) measuring 27 m long, 11 m wide and 4.3 m at the highest point were used for experiments. These systems were made of steel-structured frames with Solarig™ covered roofs and 17-mesh netting [17 apertures per every linear inch (2.54 cm) of mesh] on all sides to ensure adequate ventilation (Fig. 1A). The floor of the semi-field systems was covered with up to 60 cm of sand and was kept clear of any vegetation. The sandy floors were watered daily prior to experiments to ensure that the relative humidity remained > 70%. Temperatures inside the semi-field system varied during the months the experiments were implemented (September 2018 to March 2019) between a minimum of 18 °C at night and a maximum of 50 °C during the day. Data loggers (Tinytag View 2 Data Logger, Gemini data loggers, UK) were placed in both semi-field systems and temperature readings were taken every 30 min throughout. Each semi-field system contained a make-shift experimental hut made of angle iron frames, plywood walls and grass-thatched gable roofs with open eave gaps to mimic a traditional house in western Kenya. Each hut measured 6.5 m by 3.5 m and 2.5 m at the highest point (Fig. 1A). Between the roof and the top of the wall, all round the house was a 10-cm-wide eave gap that was representative of eave gaps of houses in local villages. The doors and windows of the huts were fully screened. Human landing catches (HLC) were conducted either outdoors, 2.5 m away from the hut, or inside at the centre of the hut.

### Mosquitoes

Female *Anopheles arabiensis* Mbita strains were obtained from the icipe-TOC mosquito insectary and were used for all bioassays. Rearing of mosquitoes in the insectary followed a standard procedure where they were reared under ambient conditions. Female mosquitoes, between 3 and 5 days old post-emergence, that had never fed on blood were selected for use in these experiments. Selection was done by holding a hand close to the outside of the cage and picking responding females out of the cage with a mouth aspirator. In each experimental night, 160 female mosquitoes were used per semi-field system. Selected mosquitoes were starved of water and glucose for 5 h prior to release in the semi-field systems to increase their host-seeking response. In each semi-field system, four cups were individually placed in all four corners and 40 mosquitoes released from each cup. Mosquitoes were dusted using a distinct fluorescent dye to distinguish them according to the four sites of release [43].

### Insecticide susceptibility of insectary mosquitoes

World Health Organization (WHO) tube tests were conducted to establish the insecticide susceptibility on the insectary-reared *An. arabiensis* females used for experiments [44]. Four tubes were used to test susceptibility of mosquitoes to 0.05% deltamethrin by exposing mosquitoes to treated papers for 1 h and then monitoring them for 24 h. Knock-down and mortality were compared to a control group that was exposed to untreated paper in parallel. Between 20 and 25 mosquitoes were used in each tube with four tubes being used to expose mosquitoes to test paper and two tubes to expose mosquitoes to the castor oil-treated control paper [45].

### Preparation of eave screens and strips

For full eave screens, burlap material from local markets was cut into bands measuring 21 m in length and 12 cm in width to ensure full coverage of the eave gaps when fixed. For incomplete screening, narrow fabric strips were cut at the same lengths but only at 5 cm width (Fig. 1B and 1C).

Emulsified transfluthrin from Bayer Global (Leverkusen, Germany) supplied at a concentration of 0.2 g/ml was used as a spatial repellent. As transfluthrin is insoluble in water [20], the emulsified concentrate simplified the preparation of a solution and the impregnation of fabric [20].

For treatment, a transfluthrin solution was prepared with approximately 1300 ml of water for each Hessian screen (12 cm wide to cover the eave gap completely) and 700 ml for each Hessian strip (5 cm wide to provide incomplete gap coverage) to ensure the fabrics are fully wetted without any dripping of the solution. Emulsified transfluthrin was added to the water to achieve a load of 2.5 g active ingredient per m^2^ of fabric. Eave screens and strips were kneaded into the transfluthrin solution until visibly saturated [20]. All fabric was dried overnight, then rolled up carefully, wrapped in aluminium foil and stored in a cold room at 4 °C. Untreated screens or strips were soaked in plain lake water and then dried and used in the same way as the treated fabric but stored at a different location.

The treated fabric was unwrapped and put along the eaves of the experimental hut every evening at 17.30 h. Care was taken to avoid contact with the walls of the hut when placing the fabric. At the end of every experimental night, the fabric was removed in the morning, rolled up, covered in aluminium and stored again in the cold room at 4 °C till evening when it was placed again. The same fabric was used for the duration of one full experiment—16 experimental nights. A fresh set of fabric was used for every subsequent 16-night experiment to ensure that the impregnated transfluthrin concentration is the same at the start of each setup. Untreated fabric was handled with a clean set of gloves to avoid cross contamination. For the application of eave screens, care was taken to ensure that the entire eave gap was covered each evening by using wires to secure the fabric (Fig. 1B). For the transfluthrin-treated strip, the fabric strips were positioned at the centre of the eave gap, ensuring an equal amount of space left open both above and below the fabric (Fig. 1C).

### Experimental procedure

Five experimental treatments, consisting of (i) no eave fabric (open eaves), (ii) untreated complete eave screen, (iii) untreated strip, (iv) transfluthrin-treated complete screen and (v) transfluthrin-treated strip, were tested in six blocks of experiments as outlined in Table 1. Due to availability of two semi-field systems, two experiments were run at the same time (experimental block). For every block two treatments were selected for the most informative comparison in a step-by-step approach to better explain observations. Human landing catches (HLC) were conducted for 4 h between 19:00 h and 23:00 h in all experiments for 16 nights. Treatments were crossed over between semi-field systems after every 4 days with a resting period of 3 days in between to allow for aeration of experimental residues [46].

**Table 1:** Summary of experimental procedures implemented as blocks of two test treatments in parallel in two semi-field systems

Experimental blocks	Location of human landing catches	Treatment in semi-field system A*	Treatment in semi-field system B*
1	Indoors	Open eave	Untreated eave strip
2	Indoors	Untreated complete eave screen	Transfluthrin-treated complete eave screen
3	Indoors	Transfluthrin-treated complete eave screen	Transfluthrin-treated eave strip
4	Outdoors	Open eave	Untreated eave strip
5	Outdoors	Untreated complete eave screen	Transfluthrin-treated complete eave screen
6	Outdoors	Transfluthrin-treated complete eave screen	Transfluthrin-treated eave strip

Indoor and outdoor human landing catches were done in independent experiments. Each block of experiments was implemented over 16 nights

*All treatments were switched between semi-field systems every 4 days, ensuring that in each experimental block the treatments were equally applied in system A and B

Four male volunteers (aged between 18 and 50 years) were rotated between the semi-field systems and experimental nights to account for variability in collection skills. The impact of the treatments was estimated separately for outdoor and indoor biting. For the simulation of outdoor biting, the volunteers sat on a chair situated 2.5 m away from the experimental hut, approximately in the middle of the semi-field system [25]. For estimates of indoor biting, the volunteers sat in the middle of the experimental hut whilst the door and windows were screened only providing entry for mosquitoes through the eave gaps.

The volunteers were checked weekly for malaria parasite infections to prevent any circulation of infected insects in the semi-field systems. In preparation for the experiments, each volunteer cleaned their feet with odourless soap up to the knee and took positions on the chair without wearing shoes. Host-seeking *An. arabiensis* females were manually aspirated as soon as they landed on the lower legs and feet of the volunteers, who were provided with torches. The mosquitoes were transferred to collection cups labelled with the hour of collection and semi-field system identifier. Protective jackets to cover heads and arms were provided while torches were used for visualization when aspirating.

### Data analysis

All analyses were carried out using R Studio statistical software from R core group version i386 3.5.1 [47]. Associations between the proportion of mosquitoes landing on volunteers (number of mosquitoes collected out of all mosquitoes released) and test treatments were analysed using generalized estimating equations (GEE) fitted with a binomial distribution with logit link function. An exchangeable correlation matrix was assumed. The unique ID of every experimental night was included in the model as repeated measure. The experimental test and the volunteer ID were included as fixed factors in the models. The experiment without any eave fabric in the system (open eave) served as reference. The model generated odd ratios (OR) and their associated confidence intervals (CI). Mean proportions and their 95% CIs were estimated based on the model by transforming the log odds (logit) of the outcome to the odds scale and from the odds scale to the probability scale. The denominator in all experiments was the total number of females released per experimental night less the mortalities prior to release. Data from indoor collections (experimental block 1–3) were analysed separately from the data from outdoor collections (experimental block 4–6). Results from experimental nights where mortality in the release cups exceeded 10% were discarded and the experimental night repeated. The point of mosquito release had no significant association with the outcome and was removed from the final models. The possible correlation between the mean air temperature (in °C) during the 4-h mosquito collection duration of every experiment and the human biting rate was explored for transfluthrin containing experiments and for non-insecticidal experiments using Pearson correlation.

### Ethical considerations

This study was approved by the Kenya Medical Research Institutes Scientific and Ethics Review committee (KEMRI-SERU), protocol no. NON-KEMRI 546.

## Results

### Insecticide susceptibility test

In total, 88 *An. arabiensis* were exposed to 0.05% deltamethrin and 48 exposed to the untreated control (castor oil) in the WHO cone assay. Twenty-four hours after the 1-h exposure, a mortality of 93.2% was found, which was corrected to 91.15% according to WHO guidelines. This suggests that the insectary-reared mosquitoes used in experiments were not fully susceptible to pyrethroid insecticides.

### Indoor impact of treatments

**Fig. 2:**
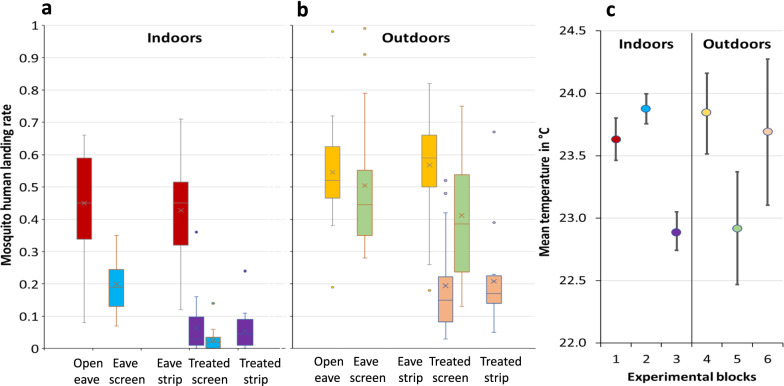
Exploration of mean proportions of *Anopheles arabiensis* attempting to bite out of all released. Box and whisker plots representing mosquito landing rates of (**a**) human landing catches (HLC) obtained indoors in the presence of the indicated conditions; (**b**) HLC obtained outdoors in the indicated conditions; (**c**) mean temperatures across all experimental blocks

In the absence of any fabric on the open eave gaps of the experimental huts, a mean of 45% (95% CI 38–52%) of all released mosquitoes was collected whilst seeking to bite the human volunteer indoors. This was similar when an untreated strip was placed at the eave gaps (Table 2; Fig. 2). Complete screening reduced the odds of the volunteer being bitten indoors by around 70% (OR 0.3), as compared to the odds of receiving a bite when the eave gaps were open (Table 2; Fig. 2).

**Table 2:** Association between proportion of mosquitoes landing on human volunteer and test treatments

	Estimated* mean proportion of released *An. arabiensis* biting (95% CI)	Odds ratio (95% CI)	*p*-value
Indoors			
Treatment			
Open eave	0.45 (0.38–0.52)	1	
Untreated complete eave screen	0.20 (0.15–0.26)	0.30 (0.20–0.47)	< 0.001
Untreated eave strip	0.43 (0.36–0.50)	0.90 (0.66–1.24)	0.520
Treated complete eave screen (block 2)	0.03 (0.01–0.06)	0.03 (0.02–0.07)	< 0.001
Treated complete eave screen (block 3)	0.06 (0.04–0.10)	0.08 (0.04–0.16)	< 0.001
Treated eave strip	0.05 (0.03–0.09)	0.07 (0.04–0.13)	< 0.001
Volunteer			
No. 1	–	1	
No. 2	–	1.06 (0.73–1.55)	0.720
No. 3	–	1.03 (0.71–1.52)	0.820
No. 4	–	0.97 (0.53–1.40)	0.910
Outdoors			
Treatment			
Open eave	0.54 (0.45–0.63)	1	
Untreated complete eave screen	0.50 (0.41–0.60)	0.85 (0.53–1.41)	0.354
Untreated eave strip	0.57 (0.48–0.65)	1.14 (0.86–1.52)	0.547
Treated complete eave screen (block 5)	0.42 (0.33–0.51)	0.62 (0.37–1.03)	0.064
Treated complete eave screen (block 6)	0.19 (0.13–0.28)	0.22 (0.12–0.38)	< 0.001
Treated eave strip	0.21 (0.14–0.30)	0.25 (0.15–0.42)	< 0.001
Volunteer			
No. 1	–	1	
No. 2	–	1.33 (0.83–2.13)	0.230
No. 3	–	0.90 (0.65–1.26)	0.592
No. 4		0.88 (0.68–1.13)	0.336

*Based on statistical model

Treating the complete screen with transfluthrin had a significant added benefit in reducing the proportion of mosquitoes seeking the host indoors. The odds of a mosquito landing on a human volunteer was 97% lower (OR 0.03) than it was when the screen was untreated based on the data from the experimental block no. 2 (Table 2, Fig. 2). Treated full screens were also tested in experimental block no. 3 and the odds of receiving a bite were similarly reduced by 92% (OR 0.08, Table 2) compared to the untreated complete screen.

Treated fabric strips were equally effective as treated full screens in reducing the odds of a mosquito attempting to bite the volunteer indoors (Table 2) when compared under the same experimental conditions (Table 1, block no. 3). No difference in landing catches was seen among the four volunteers.

### Outdoor impact of treatments

The proportion of host-seeking mosquitoes recovered outdoors through human landing catches had a mean of 54% (95% CI 45–63%) of all released, around 10% higher than what was recovered indoors in the absence of any treatments.

Open eaves (no fabric), untreated complete screen and untreated strips at the eaves resulted in the recollection of similar mean proportions of released mosquitoes from human landing collections outdoors (Table 2). In experimental block no. 5 where the experiment with untreated complete screen was run in parallel to the experiment with treated complete screen, only a slightly lower percentage in human landing was observed, from a mean of 50% of released mosquitoes landing on a volunteer in the presence of an untreated screen to an average of 42% in the presence of a transfluthrin-treated screen (Table 2). In contrast, when the treated full screen experiment was repeated in experimental block no. 6, only a mean of 19% of the released mosquitoes landed on the human volunteer. In other words, the odds of receiving a bite outdoors was reduced by 78% (OR 0.22) by the treated complete screen under the experimental conditions in block 6 (Table 2; Fig. 2) but only by 38% (OR 0.62) under the experimental conditions in block no. 5. A transfluthrin-treated strip was equally effective in reducing the proportion of host-seeking mosquitoes landing on human volunteers outdoors as the treated screen at the same test conditions (Fig. 2).

**Fig. 3:**
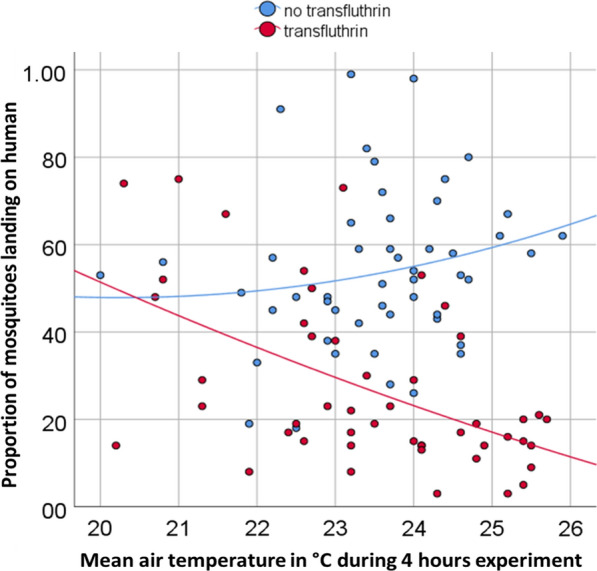
Scatter plot and trendlines exploring the relationship between mean air temperatures during the experimental runs and the proportion of released mosquitoes landing on human volunteers in the presence and absence of transfluthrin

Based on the data retrieved from the climate data loggers, only the mean temperature differed between the two experimental blocks by around 1 °C (Fig. 2). Pooling the data for outdoor experiments with transfluthrin and for experiments without transfluthrin in the semi-field system and performing a descriptive analysis, a negligible positive correlation was found between biting rates and air temperatures (*r* = 0.186, *p* = 0.099) in the absence of transfluthrin. In contrast, in the presence of transfluthrin, on either screens or strips, a negative association was found between outdoor landing rate and evening temperature (*r* = − 0.529, *p* < 0.001) with higher temperatures resulting in lower landing rates and hence higher protection (Fig. 3).

## Discussion

A simple strip of fabric treated with transfluthrin and loosely fixed around eave gaps provided similar protection against both indoor and outdoor host-seeking mosquitoes as a transfluthrin-treated complete screen; hence, transfluthrin-treated fabric strips could be substituted for complete eave screens. Untreated complete eave screening, as a physical barrier preventing mosquito entry, was highly protective indoors as shown elsewhere [11, 48–51] but expectedly did not provide any protection outdoors. Treating a complete eave screen with transfluthrin added protection indoors likely by preventing host-seeking females from entering through the remaining small gaps that resulted from fixing the screen between irregular surfaces. A complete screen provided around 70% protection from indoor biting, whilst transfluthrin-treated screens and strips provided > 90% protection from indoor biting. The corresponding outdoor protection, within 2.50 m, provided by the spatial repellency of transfluthrin was variable, preventing between 38 and 78% of the bites that would have been received by the human volunteers in the absence of a treatment. The correlated temperature data collected during the evening hours of the experiments seem to suggest that the outdoor protection by transfluthrin decreased with lower temperatures.

Temperature has been implied to play a role when working with volatile compounds such as transfluthrin [25, 52], with an indication that when temperatures are higher, volatilization is increased which could explain increased better protection against bites [25, 53]. More profoundly than for the indoor environment, the outdoor protection from transfluthrin as a spatial repellent appeared to be temperature dependent. Temperature recordings taken outdoors during this study showed a direct correlation between increasing temperatures and reduction in the proportion of mosquitoes landing on exposed persons. Landing catches in the presence of transfluthrin-treated eave fabric indicate a wide variation in the percentage protection outdoors, which is incidentally the area that would be most affected by temperature fluctuations as opposed to the indoor environment [54]. Indoor protection was not affected by the temperature variation. This might have been because house-entering mosquitoes had to pass over the transfluthrin-treated fabric at very close range [55] and/or the indoor concentration of transfluthrin might have accumulated even when released at a lower rate because of the enclosed space and no air movement inside the hut. Whether there were temperature differences between the inside of the hut and the outside is not known, since all temperature measurements were taken outdoors. Further experiments are recommended to clearly establish how temperature affects the aerial availability and concentration of transfluthrin both indoors and outdoors [53, 56, 57] to tailor recommendations for use to the most suitable eco-epidemiological settings.

This study corroborates and expands the understanding of the impact of transfluthrin-treated fabrics on open house eave gaps on indoor and outdoor-biting mosquitoes. Its use for outdoor control would be especially desirable given that there are currently no interventions targeting the outdoor biting vector population [15, 58, 59]. Results presented here confirm similar experiments implemented in Tanzania in parallel to the here-presented work [60] and demonstrate that the inclusion of a spatial repellent on the screen or strip offers protection against *An. arabiensis* biting in the outdoor environment. It is however of particular concern that its protective effect is greatly affected by temperature conditions, making future work on how to enable vaporisation of transfluthrin at lower temperatures critical [53, 57].

Notably, despite the presence of pyrethroid resistance, test mosquitoes responded to transfluthrin showing promise for it to be used as spatial repellent in areas with heightened pyrethroid resistance [44]. However, as current pyrethrum-based interventions start to be negatively affected by resistance [27], it will be vital to explore alternative non-pyrethroid compounds as spatial repellents [25].

In the absence of any treatment, higher biting rates were observed outdoors than indoors during the same time spans in the experiments, an observation that might need considered for other experimental investigations of biting behaviour in semi-field systems as it is plausible to assume that host-seeking mosquitoes accessed the human volunteer outdoors more easily. This is supported by previous observations from the same systems that the majority of released mosquitoes approach the outdoor volunteer in the first hour of release [25]. A house, where only eave gaps allow entry, still presents some physical barrier against mosquitoes compared to being outdoors. Biting indoors was observed to slowly increase hourly after the release of mosquitoes (data not shown) and might have continued beyond 23:00 h, when the experiment was stopped. The observation that host-seeking vectors managed to gain entry into the experimental hut, even when all entry points were screened, possibly through small gaps such as the grass-thatched roof, substantiates the need to continue using personal protection measures such as LLINs even as supplementary vector control tools are deployed.

The role of house modifications in reducing mosquito entry has been highlighted in many studies [3, 4, 6, 61–63] and several recommendations provided to improve house designs to reduce the vector-host contact for malaria control and elimination [10, 50]. The primary target for improvements has been the eave gaps that are usually left open in rural houses for structural reasons and improved ventilation [2, 4, 9]. While long term the improvement of house design would be desirable for all, it may not be immediately achievable because of the financial implications in resource-poor malaria-endemic communities [10]. Development of tools such as those investigated in the current study, which are protective regardless of current house designs, should be prioritized to protect people from receiving potentially infective bites. The application of treated eave strips could be a simple modification to houses, which does not require complex technical knowledge or permanent changes to the local building culture [25]. Unlike complete screening, treated eave strips would not need to be adapted for each individual house to ensure that all gaps are sealed. This would be less labour intensive and potentially less costly, assuming transfluthrin would be available for treatment. The impregnation process itself, while relatively easy, involved handling of 0.2 g/ml concentration of transfluthrin. While this concentration was below the amount considered a health risk, handling would require semi-skilled personnel for safety purposes [64]. For sustainability of this intervention, commercially produced, pre-treated, long-lasting fabric strips available on rolls for cutting to size would be most desirable.

Protection against other mosquito genera and species that are inherently outdoor- and/or indoor-biting should be explored to assess the suitability of transfluthrin-treated strips for integrated disease management [65].

## Conclusion

Presenting transfluthrin at the open eave gaps of houses provides, based on our analysis, a valuable tool under selected environmental conditions to reduce mosquito-human contact in the peri-domestic area, which includes the inside and the immediate surrounding of the house. There is no need for complete eave screens as treated fabric strips will be sufficient and can provide a simple, easy-to-handle tool for protecting people from infectious bites. However, the climatic conditions under which such a tool provides optimum protection must be investigated further and potentially incorporated into mathematical modelling approaches [66] to provide essential guidance on where and when to target this intervention. Field studies need to confirm these findings under more variable natural conditions and need to determine the range and longevity of the spatial repellent effects.

## Data Availability

The datasets used and/or analysed during the current study are available from the corresponding author on reasonable request.

## Abbreviations

HLC: Human landing catches
WHO: World Health Organisation
